# Ecology Driving Genetic Variation: A Comparative Phylogeography of Jungle Cat (*Felis chaus*) and Leopard Cat (*Prionailurus bengalensis*) in India

**DOI:** 10.1371/journal.pone.0013724

**Published:** 2010-10-29

**Authors:** Shomita Mukherjee, Anand Krishnan, Krishnapriya Tamma, Chandrima Home, Navya R, Sonia Joseph, Arundhati Das, Uma Ramakrishnan

**Affiliations:** 1 National Centre for Biological Sciences, Bangalore, Karnataka, India; 2 Ashoka Trust for Research in Ecology and Environment, Royal Enclave, Bangalore, Karnataka, India; 3 Sálim Ali Centre for Ornithology and Natural History, Coimbatore, Tamil Nadu, India; Texas A&M University, United States of America

## Abstract

**Background:**

Comparative phylogeography links historical population processes to current/ecological processes through congruent/incongruent patterns of genetic variation among species/lineages. Despite high biodiversity, India lacks a phylogeographic paradigm due to limited comparative studies. We compared the phylogenetic patterns of Indian populations of jungle cat (*Felis chaus*) and leopard cat (*Prionailurus bengalensis*). Given similarities in their distribution within India, evolutionary histories, body size and habits, congruent patterns of genetic variation were expected.

**Methodology/Principal Findings:**

We collected scats from various biogeographic zones in India and analyzed mtDNA from 55 jungle cats (460 bp NADH5, 141 bp cytochrome b) and 40 leopard cats (362 bp NADH5, 202 bp cytochrome b). Jungle cats revealed high genetic variation, relatively low population structure and demographic expansion around the mid-Pleistocene. In contrast, leopard cats revealed lower genetic variation and high population structure with a *F*_ST_ of 0.86 between North and South Indian populations. Niche-model analyses using two approaches (BIOCLIM and MaxEnt) support absence of leopard cats from Central India, indicating a climate associated barrier. We hypothesize that high summer temperatures limit leopard cat distribution and that a rise in temperature in the peninsular region of India during the LGM caused the split in leopard cat population in India.

**Conclusions/Significance:**

Our results indicate that ecological variables describing a species range can predict genetic patterns. Our study has also resolved the confusion over the distribution of the leopard cat in India. The reciprocally monophyletic island population in the South mandates conservation attention.

## Introduction

Populations of the same species living in different environments are expected to show geographic variation in genotype and phenotype. Demographic history and migration patterns over space and time can be studied through phylogeography using standing patterns of genetic variation. While many phylogeographic studies focus on single species, comparative phylogeography aims to elucidate the history and physiography of a region [1], [2]. Hence, it provides a deeper understanding of evolutionary and biogeographic processes through comparisons of congruent/incongruent patterns of distribution of variation among species and lineages [3], [4]. Most importantly, it links historical (evolutionary and biogeographic) to current (ecological) processes thus providing a temporal dimension to interpretations [4]–[6]. For example, the predominant phylogeographic paradigm for Europe and North America revolves around the Quaternary glaciations (25,000 to 10,000 years BP). Current phylogeographic patterns for many taxa in that region can be explained through range contractions into refugia (extinctions/vicariance) and post-glaciation dispersal/re-colonization events from these refugia [4], [5]. Additionally, geographical barriers such as mountain chains and rivers further explain local patterns for some taxa [6].

Biogeographically speaking, the geographic location of the Indian subcontinent is remarkable. A rich assemblage of various taxa representing major biogeographic realms (Palearctic, Africotropical, Indomalayan) occur in the subcontinent, making it a very interesting region for comparative phylogeographic studies [7], [8]. Based on paleoclimatic data, explanations for current phylogeographic patterns in India, revolving around vicariance and dispersal scenarios have been debated [9]–[11]. However, due to a combination of the paucity of genetic data, confusing, incorrect or unresolved taxonomy and the complex biodiversity and history (geological and paleoclimatic) of the region, the phylogeographic paradigm for the Indian subcontinent remains vague [10], [11]. This is borne out in a review, where phylogeographic patterns and explanations have been discussed for all major regions of the world except Asia [12]. On the other hand, it appears that for most larger-bodied mammals, (>1 kg body mass), there are few physical barriers (apart from some river systems which may act as barriers for some taxa) within the subcontinent. As a result, for such species we expect that phylogeographic patterns might be climatic and/or associated with their ecologies [10].

The family Felidae (among carnivores) is particularly well represented in India and 15 of the 36 extant species occur here [13]. Although the felid ancestor appeared approximately 10 million years ago, most species divergences in the felid phylogeny occurred within a span of the last three million years [14]. Obligate carnivory and the very rapid and fairly recent radiation in felid species has resulted in a majority of species having comparable life histories, habits and overall physiology [13], [14]. Given the apparent lack of major geographic barriers in the Indian subcontinent and the vagile nature and relatively recent evolution of felids, we expect that any difference in phylogeographic patterns among similar-sized species could be attributed to subtle and specific differences in their ecology and physiology such as tolerance to climatic factors. From a practical perspective, considerable molecular work has been conducted on the family as a whole, making it easier to generate genetic data on species within this family [14].

The jungle cat (*Felis chaus*) and the leopard cat (*Prionailurus bengalensis*) fit well within the comparative framework. The jungle cat belongs to the house cat lineage which is sister to the leopard cat lineage on the felid phylogenetic tree [14]. They are the two most common wild felids in India and often occur sympatrically. The jungle cat has morphological affinities (relatively short tail, long legs, big pointed ears) to African cats, such as serval (*Leptailurus serval*) and caracal (*Caracal caracal*), which may indicate a preference for open habitats (as opposed to closed canopy forests), whereas the leopard cat shares features (pelage color and pattern, relatively longer tail, small rounded ears) with oriental species which may indicate a similar preference for relatively more closed habitats [15]. However, despite the suggestions from morphological affinities neither cat is a habitat specialist but both are strongly associated with water [13], [15]. Though the jungle cat (average body mass in India  = 5 kg) is larger than the leopard cat (average body mass in India  = 3 kg), there is an overlap in body mass, especially of female jungle cats and male leopard cats [15], [16]. The currently accepted distributions of the two felids show them to be widespread and continuously distributed within India [13], [15], [17], however there remain ambiguities in leopard cat distribution and their presence in Central India is questioned [15], [16]. Given the accepted distribution for the two species and similarities in habits and body size, we hypothesize that they would have congruent patterns of genetic variation and structure, despite potential differences in their ecology.

In this paper, we investigated the comparative phylogeography of jungle and leopard cats within the Indian subcontinent. We mainly used non-invasive samples (scat) collected from natural habitats and mitochondrial DNA sequence analysis. Following Moodley and Bruford (2007) [18], we tested explanatory variables which could best explain the partitioning of genetic variation in both species, including latitudinal ranges, subspecific taxonomy and biogeographic classes, using the biogeographic classification for India by Rodgers and Panwar (1988) [19]. We conducted a niche model analysis using bioclimatic (derived from mean and extremes of temperature and rainfall data) variables and geo-referenced locations (latitude-longitude) for leopard cats (museum samples, historical records, ad-hoc records authenticated from photographs and current sampling), to explain their genetic structure. We restricted this to leopard cats since their genetic diversity and structure required further scrutiny. Finally, we attempted to explain the existing genetic pattern and spatial distribution of leopard cats in India through current and historical climatic conditions for the region and explored how they support the proposed vicariance hypothesis.

## Materials and Methods

### Sample collection

**Figure 1:**
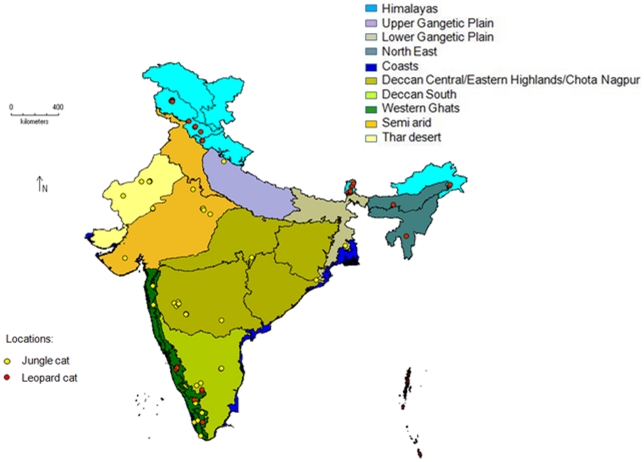
Locations of scats collected in various biogeographic zones, used in the study. Red circles: leopard cat (*Prionailurus bengalensis*) scats, yellow circles: jungle cat (*Felis chaus*) scats.

We collected scats from various biogeographic zones [19] which represent major habitat ecoregions in India from where the two species have been recorded. The regions covered were the Himalayas, Upper and Lower Gangetic plains, North-East India, Thar Desert, Semi-arid zone, Deccan Central (Central Plateau, Eastern Highlands and Chotta-Nagpur), Deccan South and Western Ghats (Figure 1). Apart from these, scats were obtained from captive individuals in zoos located within these biogeographic zones.

Since some of the broad biogeographic zones could be composed of several forest types/habitats (e.g. the Semi-arid zone would have riverine tracts, dry deciduous forests, thorn scrub), within each of the biogeographic zones, we sampled extensively to cover the various habitats present. We collected scats by walking through the habitats as well as by driving slowly (<20 km/hour) along dirt tracts and roads, wherever possible, to cover as much area as possible. Scats from natural habitats were collected when encountered and stored in vials containing 90–100% alcohol. Latitudinal and longitudinal coordinates were recorded using a hand held Geographical Positioning System (GPS) unit. Notes on date of collection and other important features such as presence of tracks were also recorded. A total of 543 scats were collected from all biogeographic zones. We surveyed several localities/districts in each eco-region to avoid sampling related or same individuals.

### Laboratory methods

Since scats collected in natural habitats could belong to several carnivores, we had to first assign scats collected to the species of our interest. For this we used a PCR-RFLP protocol [20] based on the 16 s rRNA gene for a certain proportion of scats, until the required number of scats for each species from each region was obtained. To avoid sampling the same or related individuals (since cats show female philopatry), as far as possible we selected scats that were located in different districts within a biogeographic zone. Scats that were within 5 km from each other were included only if the sequences generated from them differed from each other (were separate haplotypes). We had a total of 40 leopard cats and 55 jungle cat scats for further analysis.

Primers were designed using existing sequences for the two species as well as from house cat sequences downloaded from NCBI. Initially, we designed primers for the Control Region using domestic cat sequences, since sequences for this region for our study species were not available. However, two sets of primers that amplified a total of 377 bp, worked on our species of interest but were in the non-variable portions of the Control Region. We sequenced 10 individuals of jungle cat from various parts of its distribution (North-East India, Upper Gangetic Plains, South and Central Deccan Peninsular, Western Ghats, Lower Gangetic Plains and one from Iraq) using these primers and found no difference between them. Hence we selected the next most variable regions, NADH5 and cytochrome b, based on information from Johnson and O'Brien (1997) [21]. Within these regions we designed several primers and sequenced several individuals of both species before choosing primers that amplified the most variable portion of these regions. We initially designed a primer set for the cytochrome b region for jungle cats based on house cat sequences. However, we later designed another primer set for the same region which amplified a longer portion which was then used for both species. Since we had already generated sequences for several jungle cat individuals using the previous primer pair, we truncated the jungle cat sequence length for the cytochrome b region. Hence the final length of jungle cat cytochrome b region is smaller than that of leopard cat although they are from corresponding regions. The final set of primers we used (Table 1) were for regions of NADH5 (362 base pairs for leopard cat and 460 base pairs for jungle cat) and cytochrome b (202 base pairs for leopard cat and 141 base pairs for jungle cat) genes. Since most of our work was on non-invasive samples (scat) which are relatively poor sources of DNA, we had to amplify several small fragments of DNA to obtain the total length required (564 base pairs for leopard cat and 601 base pairs for jungle cat). Each of our primer pairs amplified between 100 and 200 base pairs (Table 1). We standardized primer annealing temperatures on blood samples obtained from captive individuals.

**Table 1:** Details of primers used in the study.

Gene	Name	Sequence (5′….3′)	Species	Amplicon length	Annealing temperature
NADH5	JCND5_159F JCND5_159R	CCTATGCCTTTACCATCAGCA GTGCCACGGGAATGAAGAT	Jungle cat	98 bp	59°C
	JCND5_210F JCND5_210R	CTGTGGCACTTTTCGTCA TAAAGCGGCAGTGTTTGC	Jungle cat	186 bp	55°C
	JCND5_4 F JCND5_4 R	ATCCTCTACAACCGCATTGG AGACAGGAGTTGGGCCTTCT	Jungle cat and Leopard cat	176 bp	59°C
	LepcatND5 F LepcatND5 R	GACCCATATATCAACCGA GCGTTTGAGTTAGTAAGG	Leopard cat	186 bp	55°C
Cyt b	HCJC FHCJC R	ATCTCAGCCTTAGCAGCA TTGTCTGGGTCTCCTAGC	Jungle cat and Leopard cat	141 bp202 bp	50°C
	LepcatCytb2 F LepcatCytb2 R	CTGTCTATACATGCACGT TGGCTTTGTCTACTGAGA	Leopard cat	239 bp	56°C
	LepcatCytb3 F LepcatCytb3 R	CATCTTAGGCCTTCTAGT GGAGGATTGGAATGATTG	Leopard cat	236 bp	52°C

DNA was extracted using QIAmp (QIAGEN) tissue and stool kits following the manufacturer's protocols with slight modifications [22]. All extractions were carried out in a PCR-free environment to decrease chances of contamination. Extractions from scat and blood were carried out in separate rooms to minimize chances of contamination from blood to feces. Since the samples were mostly fecal, we included controls with all extractions to monitor contamination. PCR amplifications were carried out in 10 µl PCR reactions using a PCR master mix (QIAGEN, Inc.), 4 µg Bovine Serum Albumin (Sigma) and 2 µM primers using the following program: Initiation at 94°C for 10 min, followed by 94°C for 30 s, 49–60°C (annealing, depending on the primer pair, see Table 1) for 45 s, 72°C for 50 s, followed by 10 min at 72°C, repeated for 59 cycles. All PCR reactions included controls to monitor contamination as is required with non-invasive samples.

Additionally, we designed primers using existing leopard cat cytochrome b sequences [23] and compared a total of 575 bp (two new fragments of 239 bp and 236 bp along with 100 bp of the previously sequenced portion) of Indian sequences to sequences from East and South East Asia. Only 5 samples (one each from the North East, Eastern Himalayas and Western Himalayas and two from Western Ghats) were used for this. The PCR program used was the same as above with annealing temperatures of 56°C for the primer pair Lepcatcytb2 and 52°C for Lepcatcytb3. This was done only for the leopard cat since sequences of populations from outside India were available only for this species [23].

PCR products were visualized on a 2% agarose gel and products were purified using Exonuclease-Shrimp Alkaline Phosphatase (0.7∶1 ratio) mixture (USB Corporation) prior to sequencing. Products were sequenced in both forward and reverse directions using the ABI Big Dye Terminator sequencing kit in an ABI 310 automated sequencer (Applied Biosystems).

We randomly picked some scats (that showed new haplotypes after sequencing) and repeated the entire process from extraction to sequencing, to check the validity of new haplotypes.

### Sequence data submission

All new data has been deposited in GenBank (Jungle cat NADH5: GU561646-GU561700; Jungle cat Cytochrome b: GU561701-GU561755; Leopard cat NADH5: GU561756-GU561795; Leopard cat Cytochrome b: GU561796-GU561806, GU561808-GU561814, GU561816-GU561837). Details of sample identities, their locations and accession numbers are provided in Table S1.

### Analyses

Sequences were aligned using the program MEGA [24]. Using combined NADH5 (303 bp) and cytochrome b (141 bp) regions we selected unique haplotypes for both species. With the fishing cat as outgroup, we constructed phylogenetic trees using the software PAUP* (version 4.0) [25] and the best-fit model for nucleotide frequencies, transition-transversion ratio and nucleotide substitution by the Akaike Information Criterion (AIC) [26] in ModelTest (version 3.8) [27], [28]. We used the Neighbor Joining (NJ) [29] method based on Jukes-Cantor distances with 1000 bootstrap replicates as well as the Maximum Likelihood (ML) [30] method with 500 bootstrap replicates based on a heuristic search.

The NADH5 and cytochrome b regions were combined for each species (jungle cat: 460 bp NADH5, 141 bp cytochrome b, leopard cat: 302 bpNADH5, 202 bp cytochrome b) and genetic structure was assessed through median-joining haplotype networks [31] using the program NETWORK (version 4.5.1.0; http://www.fluxus-engineering.com).

For leopard cats, a separate Maximum-Likelihood tree based on a heuristic search and 500 bootstrap replicates, as well as a Neighbor-Joining tree with Jukes-Cantor distances and 1000 bootstrap replicates were built using PAUP* with 575 bp of cytochrome b and sequences included from the earlier study [23], using the fishing cat as outgroup. A median-joining haplotype network for the same dataset was also built using the program NETWORK.

Intra-population measures of diversity (number of haplotypes, gene diversity, nucleotide diversity (π), average pairwise difference (θπ), and number of segregating sites (θs)) were calculated for each species using the software ARLEQUIN (Version 3.1) [32]. Genetic structure for the two species was investigated through *F*_st_ with pairwise differences, using the analysis of molecular variance (AMOVA). This was done for all categories of explanatory variables including biogeographic classes, latitudinal ranges and subspecific classification.

We followed Pocock's 1939 [16] taxonomic classification of subspecies for jungle cat and leopard cat. He described four subspecies of jungle cat in India based on morphological characters. These were *F. c. affinis* (Himalayas), *F. c. kutas* (northern peninsular India), *F. c. prateri* (Thar desert) and *F. c. kelaarti* (southern India). Pocock 1939 [16] split leopard cat populations in India into two subspecies. He called the Himalayan ones *P. b. horsfieldi* and clubbed the North Eastern and South Indian populations into one called *P. b. bengalensis*. For latitudinal grouping we used classes of 10°N–19.9°N, 20°N–28.9°N and 29°N-35°N that broadly defined south, central and northern India respectively.

Tajima's D [33] and Fu's Fs [34] were computed with 1000 simulations, to test for neutrality and demographic history and we used the mismatch analysis for both species, to estimate demographic parameters of past population expansions [35] These parameters (τ, *θ*_0_, and *θ*_1_) estimated by a generalized nonlinear least-square approach with confidence intervals computed using a parametric bootstrap approach were obtained using ARLEQUIN (Version 3.1) [32]. The population expands from an initial θ_0_ to θ_1_ in τ units of mutational time (τ is also the mode of the mismatch distribution), where θ = 2N_e_ * µ (N_e_ is the effective population size for females and µ is mutation rate per generation for the sequence studied). Time since expansion (in generations) can be calculated as t = τ/2 µ [32], [36]. We calculated time since expansion for the jungle cat, with 601 bp of sequence from NADH5 and cytochrome b, an estimated mutation rate of 1.3%/bp/million years, (combined rate of cytochrome b (1.38% MY) [37] and NADH5 (1.22% MY) [38]) and generation time of a year.

Furthermore, we tested if geographic and genetic distances are correlated (isolation by distance) in the two species, using the biogeographic and taxonomic classification for grouping populations of jungle cat and only the taxonomic classification for leopard cats since the biogeographic grouping in this species had just two populations. We generated geographic distances between individuals using the program Geographic Distance Matrix Generator, (Version 1.2.3) [39] and took the average distance of individuals of one population from individuals of another. We tested for the association between pairwise geographic and genetic distances (*F*_st_) by conducting a Mantel test using the IBD software (Version 1.53) [40] without log transformations and with 10,000 randomizations for obtaining values for statistical significance.

To explain the genetic pattern observed in leopard cat we explored how current climatic patterns could influence its distribution, using niche-model analysis. Geo-referenced (latitude and longitude) unique locations of leopard cat (n = 140) were obtained from museum specimens across the globe, from the current study, from literature [16], [23], [41], as well as locations reported by others authenticated with photographs. From details on the specimen vouchers and labels, we verified that none of these records were duplicated (since many of the specimens in Pocock's (1939) [16] literature are specimens housed in the Bombay Natural History Society, and the Natural History Museum (London) collections. Museum sample records were obtained by correspondence, visits to some museums, from the Global Biodiversity Information Facility (Accessed through GBIF Data Portal, www.gbif.net,on 15^th^ December 2008) and from the Mammal Networked Information System (Accessed through the MaNIS portal http://manisnet.org, on 15^th^ December 2008). We obtained location records from the Bombay Natural History Society, Natural History Museum (London), Smithsonian National Museum of Natural History (Washington), Field Museum, (Chicago), Los Angeles County Museum of Natural History (Los Angeles), University of Kansas Biodiversity Research Centre (Kansas), Swedish Museum of Natural History (Stockholm), Museum für Naturkunde (Berlin), California Academy of Sciences and the University of Michigan Museum of Zoology (Michigan).

We extracted bioclimatic data from the WORLDCLIM data set (Version 1.4, http://www.worldclim.org/bioclim.htm) [42] for 2.5 min intervals. This dataset ranging over a 50 year period (1950 to 2000) and collected over several globally located weather stations, uses annual trends, extremes and seasonality of temperature and precipitation to derive biologically meaningful variables [43]. 19 bioclimatic variables (annual mean temperature, mean monthly temperature range, isothermality (2/7 * 100), temperature seasonality (standard deviation of monthly temperature *100), maximum temperature of the warmest month, minimum temperature of the coldest month, temperature annual range (5–6), mean temperature of wettest quarter, mean temperature of driest quarter, mean temperature of warmest quarter, mean temperature of coldest quarter, annual precipitation, Precipitation of wettest month, precipitation of driest month, precipitation seasonality (CV), precipitation of wettest quarter, precipitation of driest quarter, precipitation of warmest quarter, precipitation of coldest quarter) were used for the initial analyses.

Since correlation between variables can lead to model over-fitting [44] we computed Pearson's correlation coefficient (*r*) between each pair of variables, using SPSS 16.0 statistical software. The correlation was done by extracting climatic information from 400 unique, randomly generated points within the global spread of the distribution of the two cats, using DIVA-GIS (version 7.1.7.2, http://www.diva-gis.org). We selected 8 variables that were not highly correlated to each other, using *r* = 0.7 as the cut off. These were, maximum temperature of the warmest month (Bio 5), temperature annual range (Bio 7), mean temperature of the driest quarter (Bio 9), mean temperature of the coldest quarter (Bio 11), precipitation of wettest month (Bio 13), precipitation seasonality (Bio 15), precipitation of warmest quarter (Bio 18) and precipitation of the coldest quarter (Bio 19). These variables were selected over others because they included climatic extremes (Bio 5, Bio 7) and others that we felt were biologically more meaningful. We chose extreme climatic factors since these are perhaps better ecological indicators of species distributions and range limits than averages.

We developed distribution models using two distinct techniques, the Maximum Entropy [45] which is a relatively more complex and robust model [46] and BIOCLIM [47] which is simpler in its calculations. We used the software MaxEnt (version 3.3.2) and DIVA-GIS (version 7.1.7.2, http://www.diva-gis.org) to construct the models. The maximum entropy approach uses a machine-learning algorithm which assumes a uniform probability distribution for presence in the region of interest, subject to certain constraints provided by the distribution of known presences across environmental factors. The model is fit and improved over several iterations. For constructing the model it uses presence data, a background of randomly selected points that it creates from the region of interest, and the climatic features for each point. The final map predicts suitability of habitat as a probability of presence where zero indicates not suitable and one is highly suitable [45], [46].

In contrast BIOCLIM is based on a heuristic search method that measures environmental values from known locations of species (presence-only data) to identify other areas with environmental ranges that are encompassed within those envelopes. Envelopes are computed for each climatic feature/variable with the maximum and minimum values of the presence points. Results are presented as habitat suitability which is derived from the percentile of points falling within envelopes. Regions with points that fall within all envelopes constitute the most suitable habitat [47].

We used the following settings for the MaxEnt model: Ten replicates in batch mode with auto features (where feature types are selected by the program based on the training sample size), jackknife tests, logistic output format, random test percentage  = 25, replicate run type  =  crossvalidate, regularization multiplier  = 1, maximum iterations  = 500, convergence threshold  = 0.0001 and maximum number of background points  = 10,000. For the BIOCLIM model, we used the sample point option to generate random pseudo-absence points with ten replicates (i.e. 140 random points were generated 10 times, independently). The training (75% of all presence points randomly drawn) and test data (25% of total points randomly drawn) were also selected through 10 separate replicates using the same option. The training data was run as a batch file using the BIOCLIM option after selecting the climatic variables of interest. The envelopes were set at the 0.025 percentile cut-off level to exclude extreme climatic values (i.e. the envelopes encompassed variation for climatic variables corresponding to locations, within the 97.5th percentile and all values falling outside this were excluded as outliers). Further, using the evaluation option the training replicates were tested with the test replicates to generate AUC values.

We evaluated and compared models from the two distinct approaches using the Receiver Operating Curve (ROC)/AUC (Area Under Curve) statistics. The ROC curve plots the ability of the model to predict true presences (specificity) against its false positives (error of commission), across all possible thresholds. The AUC is then calculated from the ROC plot as a threshold-independent measure of the model's performance. Values for AUC range from 0 to 1 where one indicates a perfect prediction, 0.5 a prediction that would be no different from random and all values less that 0.5 would indicate poor prediction [48].

For the final maps of predicted habitat suitability from MaxEnt and BIOCLIM all 140 unique presence data points for leopard cat were used. In BIOCLIM all variables are given equal weights and hence it does not have a variable weighting function, while in MaxEnt variables are weighted (measured as gain) according to the way they influence model fit. Hence the contribution of each variable to the final prediction was determined only for MaxEnt. Gain can be explained as the contribution by the variable to model fit where an increase in gain due to the variable leads to a better fit. In MaxEnt, a jackknife test was performed on both training and test data as well as for AUC on test data. This test estimates the gain for individual variables in isolation, as well as the loss in overall model gain when each variable is omitted. It also reports the increase or drop in test AUC with the inclusion and exclusion of the variable [45]. As an additional test of variable importance, we mapped the presence locations of leopard cats with each climatic variable.

## Results

A total of 543 scats were collected from all major biogeographic zones within the country. Our laboratory-based species identification of scat samples revealed that jungle cat scats were found within all biogeogoraphic zones sampled, but were rare in the northern part of the country (Himalayas, North East and foothills) and hence for the biogeographic grouping we had to pool the Upper Gangetic Plains and North East samples since they are neighboring zones. On the other hand leopard cat scats were found only from the Himalayan zone, North East India and the Western Ghats. In all we had a total of 55 jungle cat scats and 40 leopard cat scats (Figure 1). In the case of jungle cats there were 16 cases (pairs) where scats were less than 10 km apart (these were from the Arid and Central Deccan zones). However, except for three pairs (5 scats) that were within this distance from each other, the remaining 14 pairs (of 14 scats) were all from different individuals (their sequences differed). The five scats were from the arid zone and were on average 6 km apart. The scats that belonged to different individuals were from the Arid, Central Deccan and Semi-arid zones and were between 3 km to 5 km apart. In the case of the leopard cat there were 16 pairs (8 pairs of 5 scats from the Western Himalayas and 8 pairs of 7 scats from the Western Ghats) that were less than 10 km apart and two of these belonged to different individuals (inferred from haplotypes), while the remaining were on average 8 km apart in the Western Himalayas and 7 km apart in the Western Ghats.

The set of primers we used (Table 1) were for regions of NADH5 (362 base pairs for leopard cat and 460 base pairs for jungle cat) and cytochrome b (202 base pairs for leopard cat and 141 base pairs for jungle cat) genes.

Our data revealed a total of 33 haplotypes with 33 polymorphic sites (28 transitions and 6 transversions) for jungle cat and 8 haplotypes with 11 polymorphic sites (9 transitions and 2 transversions) for leopard cat. Overall genetic diversity in the jungle cat was higher than in leopard cat which is summarized in Table 2.

**Table 2:** Overall genetic diversity for jungle cat and leopard cat populations in India.

	Jungle cat	Leopard cat
N	55	40
Base pairs	601	564
Haplotypes	33	8
Gene Diversity	0.976+/−0.008	0.793+/−0.039
Nucleotide Diversity (π)	0.007+/−0.003	0.005+/−0.003
θ π	3.452+/−1.985	3.074+/−1.814
θ s	7.212+/−2.283	2.601+/−1.061
Tajima's D (*P*)	−1.725 (0.02)	0.648 (0.781)
Fu's F (*P*)	−26.04 (0.00)	0.813 (0.657)

### Phylogenetic analyses and haplotype networks

We built combined trees for jungle cat and leopard cat with 444 bp of NADH5 and cytochrome b, since the regions sequenced for these genes did not coincide completely between the two species. ModelTest revealed that the General Time Reversible with Gamma distribution (GTR+G) model best fit the sequence data, with the following settings: number of substitution types = 6, user-specified substitution rate matrix = (3934011648.0000 49585004544.0000 2565318656.0000 0.0218 49585004544.0000), assumed nucleotide frequencies: A = 0.31680 C = 0.26430 G = 0.12830 T = 0.29060, shape parameter (alpha)  = 0.3442, number of rate categories = 4, assumed proportion of invariable sites = none, distribution of rates at variable sites = gamma (discrete approximation), representation of average rate for each category = mean.

**Figure 2:**
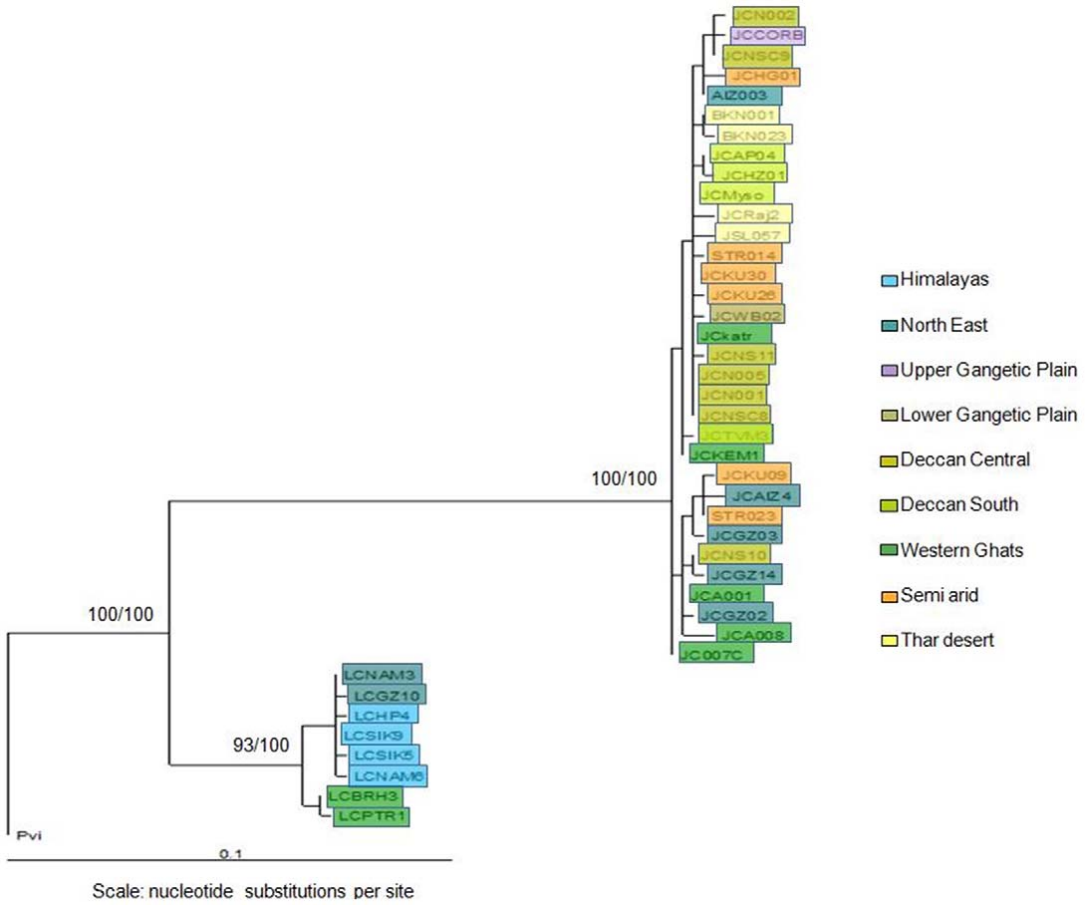
Phylogenetic tree of Indian populations of jungle cat and leopard cat. Phylogenetic relationships using mtDNA sequences of 55 jungle cats (*Felis chaus*) and 40 leopard cats (*Prionailurus bengalensis*) (303 bp NADH5, 141 bp cytochrome b) from India. The trees are rooted with fishing cat (*Prionailurus viverrinus*). The Maximum-Likelihood (-ln L = 970.60524) tree was constructed using PAUP*, heuristic search with 500 replicates, the GTR+G (General Time Reversible with Gamma distribution) model and empirically derived nucleotide frequencies. The Neighbor-Joining tree was constructed with PAUP* using Jukes-Cantor distances and 1000 bootstrap replicates. The tree presented is the Maximum Likelihood tree. The Neighbor-Joining tree showed an identical topology. Numbers indicate bootstrap support in percent (ML/NJ).

Both ML and NJ trees revealed the same overall pattern, for jungle cats and leopard cats. Jungle cats within India form a single shallow, unresolved clade. Leopard cats within India separate out into two clades, the North (Himalayan/North East) and the South (Western Ghats). The split between the Himalayan/North East and Western Ghats populations of leopard cat had a bootstrap support of 93% for the ML and 100% for the NJ tree (Figure 2).

**Figure 3:**
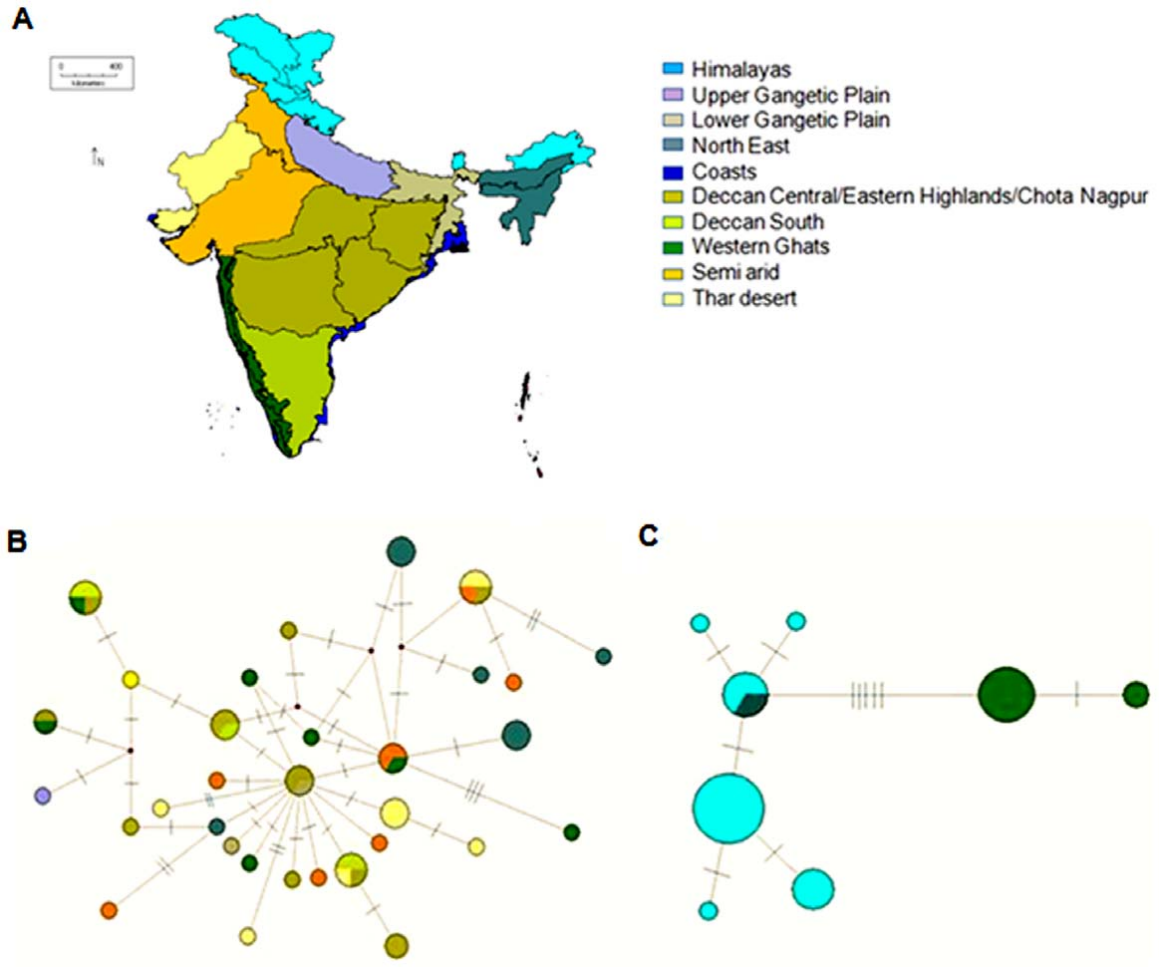
Haplotype networks. **3A** Biogeographic regions of India corresponding to haplotypes. **3B**. Median Joining haplotype network for 55 jungle cats (*Felis chaus*) with 460 bp NADH5 and 141 bp cytochrome b. **3C**. Median Joining haplotype network for 40 leopard cats (*Prionailurus bengalensis*) with 362 bp NADH5, 202 bp cytochrome b. Bars on branches denote number of substitutions between connected haplotypes. Size of circle denotes number of individuals in the haplotype. Small circles are missing haplotypes.

Haplotype networks were built with 601 bp (460 bp of NADH5 and 141 bp of cytochrome b) sequence for jungle cat and 564 bp (362 bp of NADH5 and 202 bp of cytochrome b) for the leopard cat. Figures 3A and 3B reveal relatively low structure in the jungle cat, while the leopard cat network (Figures 3A and 3C) shows a clear difference between the Himalayan/North East India and Western Ghats.

**Figure 4:**
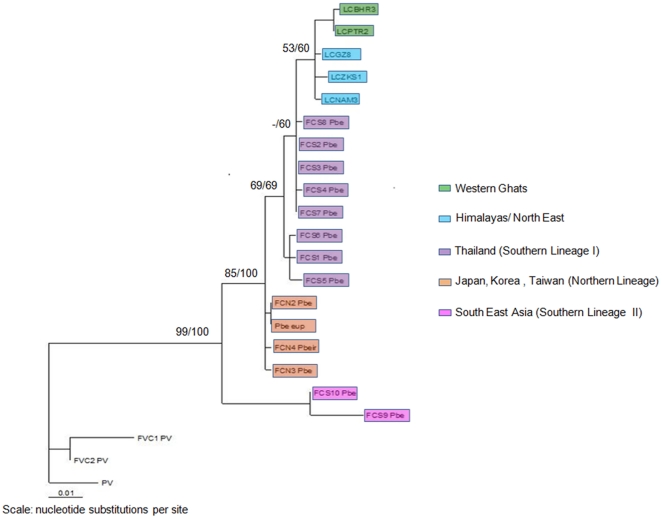
Maximum Likelihood tree of cytochrome b for leopard cats, rooted with fishing cat. Phylogenetic relationships of the global population of leopard cats (*Prionailurus bengalensis*) using 575 bp of cytochrome b sequence. The tree is rooted with fishing cat (*Prionailurus viverrinus*). The Maximum-Likelihood (-ln L = 1148.87045) tree was constructed using PAUP* and heuristic search with 500 replicates and the GTR+I (General Time Reversible with Invariable sites) model and empirically derived nucleotide frequencies. The Neighbor-Joining tree was constructed with PAUP* using Jukes-Cantor distances and 1000 bootstrap replicates. The tree presented is the Maximum Likelihood tree. The Neighbor-Joining tree showed an identical topology. Numbers indicate bootstrap support in percent (ML/NJ). Sequences outside India were generated by Tamada et al. (2008) [20].

**Figure 5:**
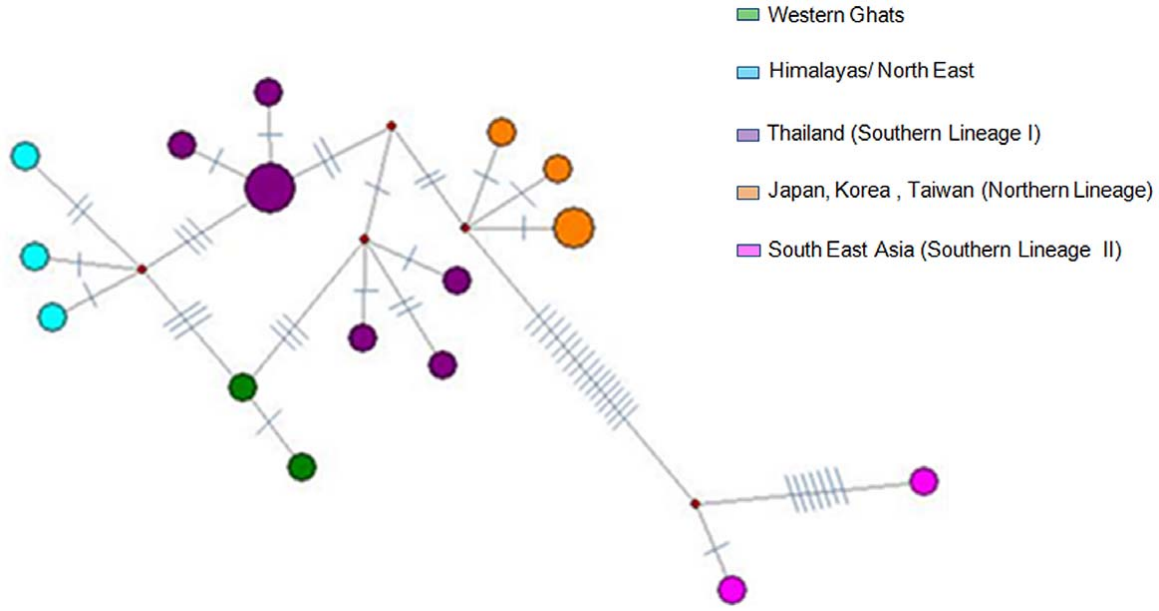
Median-Joining haplotype network for leopard cats (*Prionailurus bengalensis*) with cytochrome b (575 bp). Bars on branches denote number of substitutions between connected haplotypes. Size of circle denotes number of individuals in the haplotype. Small circles are missing haplotypes.

We further analyzed a more global leopard cat dataset which included Indian, East and South East Asian samples. We restricted this only to the leopard cat since we had sequences of leopard cat from outside India available from a recent publication [20]. Sequences of jungle cat from outside India are not available and so we could not do a similar analysis for this species. For this leopard cat sequence data the General Time Reversible with Invariable sites (GTR+I) model was selected by the AIC using ModelTest. The ML and NJ tree for 575 bp of cytochrome b revealed that the Indian populations were close to the Thailand population. However, both trees showed polytomy (low resolution) between the Thailand (Southern Lineage I) and Korean/Japanese/Taiwanese (Northern Lineage) populations and between the Thailand and Indian populations (Figure 4). The haplotype network for the cytochrome b region (575 bp) of leopard cat sequence showed results that were similar to the trees where the relationship between the Indian and Thailand populations were not clearly resolved, with several missing haplotypes (Figure 5) potentially due to inadequate sampling in areas like South China, Myanmar and Orissa (India).

### Genetic diversity and structure

All measures of genetic diversity (gene diversity, number of haplotypes, nucleotide diversity and segregating sites) were higher on an average for the jungle cat compared to the leopard cat (Table 2). AMOVA results for the jungle cat using various models of classification yielded similar results, with *F*_ST_'s of 0.10, *P*<0.05 (latitudinal classification), 0.11, *P*<0.05 (taxonomic) and 0.12, *P*<0.05 (biogeographic) (Table 3). However, for the leopard cat a higher differentiation between groups was obtained using the biogeographic approach that generated a *F*_ST_ of 0.86 (*P*<0.05) as compared to a *F*_ST_ of 0.32 (*P*<0.05) for the taxonomic categorization (Table 3).

**Table 3:** AMOVA results for both species given different explanatory variables.

Species	Biogeographic/ecoregion	Taxonomic	Latitudinal ranges
Jungle cat(*n* = 55)	0.12*	0.11 *	0.1 *
Leopard cat(*n* = 40)	0.86 *	0.32 *	-

*significance at *P*<0.05.

Using the biogeographic classification for the jungle cat, the maximum differentiation was seen between the South Deccan and Upper Gangetic Plains/North East populations which showed an *F*_ST_ of 0.29 (*P*<0.05) followed by the South Deccan and Semi arid populations (*F*_ST_ = 0.23, *P*<0.05) (Table 4). Differences were also seen between the Central Deccan/Lower Gangetic Plains and Upper Gangetic Plains/North East (*F*_ST_ = 0.17, *P*<0.05), Western Ghats and Thar (*F*_ST_ = 0.12, *P*<0.05), South Deccan and Thar (*F*_ST_ = 0.19, *P*<0.05), Central Deccan/Lower Gangetic Plains and Thar (*F*_ST_ = 0.1, *P*<0.05) and Upper Gangetic Plains/North East and Thar (*F*_ST_ = 0.16, *P*<0.05) populations (Table 4).

**Table 4:** AMOVA results: jungle cat with the Biogeographic/ecoregion approach.

Biogeographic region	W Ghats(*n* = 7)	S. Deccan(*n* = 8)	C. Deccan-Lower Gangetic Plain (*n* = 13)	Semi Arid(*n* = 8)	Upper Gangetic Plain North-East(*n* = 10)
S. Deccan	0.09				
C. Deccan-Lower Gangetic Plain	-0.01	0.03			
Semi Arid	0.06	0.23*	0.08		
Upper Gangetic Plain-North East	0.09	0.29*	0.17*	0.04	
Thar (n = 9)	0.12*	0.19*	0.10*	0.03	0.16*

*significance at *P*<0.05.

The taxonomic classification for jungle cat showed significant structuring between, *F. c. affinis* (Himalayan population) and *F. c. kelaarti* (South Indian population) with (*F*_ST_ = 0.2, P<0.05), *F. c. affinis* and *F. c. kutas* (Central Indian population) (*F*_ST_ = 0.12, P<0.05), *F. c. prateri* (Thar desert population) and *F. c. kelaarti* (*F*_ST_ = 0.14, P<0.05) and *F. c. prateri* and *F. c. affinis* (*F*_ST_ = 0.16, P<0.05).

The latitudinal gradient classification showed significant structuring between all classes (10°N–19.9°N and 20°N–28.9°N: *F*_ST_ = 0.05, P<0.05; 10°N–19.9°N and 29°N–35°N: *F*_ST_ = 0.19, P<0.05; 20°N-28.9°N and 29°N-35°N: *F*_ST_ = 0.12, P<0.05).

The biogeographic/ecoregion classification for the leopard cat revealed *F*_ST_'s of 0.9 and 0.91 (*P*<0.05) between the Himalaya and Western Ghats and the North East and Western Ghats populations, respectively (Table 5). The Himalayan and North East populations showed an *F*_ST_ of 0.3 (*P*<0.05). The taxonomic grouping for leopard cats showed a significant *F*_ST_ of 0.32 (*P*<0.05) between *P. b. horsfieldi* and *P. b. bengalensis*.

**Table 5:** AMOVA results: leopard cat with Biogeographic/ecoregion approach.

Biogeographic region	Himalaya(*n* = 19)	North East(*n* = 9)
North East	0.30*	
Western Ghats(*n* = 12)	0.90*	0.91*

*significance at *P*<0.05.

**Figure 6:**
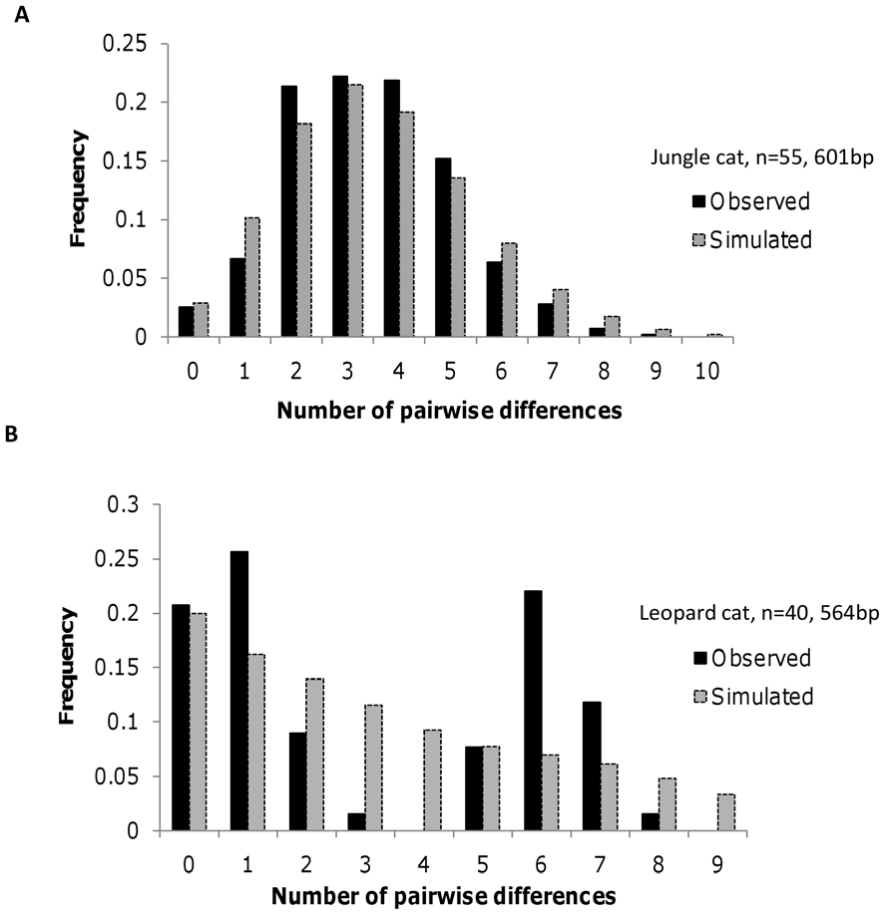
Mismatch distribution analysis of mitochondrial DNA. **6A**. Jungle cat (*Felis chaus*) (n = 55 individuals, 601 bp consisting of 460 bp of NADH 5 and 141 bp of cytochrome b). **6B**. Leopard cat. (*Prionailurus bengalensis*) (n = 40 individuals, 564 bp consisting of 362 bp of NADH 5 and 202 bp of cytochrome b).

Both Tajima's D (−1.725 *P*<0.05) and Fu's F (−26.04, *P<*0.05) values were negative and significant in jungle cat implying an excess of rare haplotypes (Table 2). On the other hand, the pooled leopard cat data showed non-significant, low positive values for Tajima's D and Fu's F (Table 2). Mismatch analyses were unimodal, with an average of 3.5 pairwise differences for jungle cat and bimodal (with peaks at 1 and 6 pairwise difference) for the leopard cat (Figure 6A and B; Table 6).

**Table 6:** Fitting of mismatch distribution to a sudden expansion model.

Taxon	Mismatch mean	SSD(*P*)	τ(95% CI)	θ_0_(95% CI)	θ_1_(95% CI)	Harpending's raggedness index (*P*)
Jungle cat	3.452	0.004(0.136)	3.543(2.605–4.240)	0.000(0.000–0.693)	99999.0(20.133–99999)	0.037(0.094)
Leopard cat North India	0.942	0.015(0.146)	1.084(0.492–2.291)	0.000(0.000–0.028)	99999.0(1.874–99999)	0.138(0.100)
Leopard catSouth India	0.303	0.236(0.142)	2.982(0.000–7.982)	0.900(0.000–6.589)	3.600(0.908–99999)	0.247(0.402)

Since a unimodal peak of the mismatch distribution and significant, negative values of Tajima's D and Fu's F indicate population range expansion, we also estimated the time since expansion for the jungle cat. Time (in generations) since expansion can be calculated as τ/2 µ [32], [37], [38] where τ corresponds to the mode of the mismatch distribution and µ is the mutation rate per generation for the sequence under study. The generation time for jungle cats was assumed to be one year. Based on a mutation rate of 1.3%/bp/million years (combined cytochrome b (1.38% MY) [37] and NADH5 (1.22% MY) [38], 601 bp of sequence (NADH5 and cytochrome b) and τ values between 2.605 and 4.24 (Table 6), our estimates of the range expansion for jungle cat in India dates to the mid Pleistocene (166,709 to 271,342 years ago).

Jungle cats showed significant isolation by distance for both the biogeographic (6 groups, r = 0.59, p<0.05) and taxonomic (4 groups, r = 0.95, p<0.05) groupings. Only the taxonomic grouping could be used for leopard cats for testing isolation by distance since there were only two groups in the biogeographic grouping for this species. The taxonomic grouping showed a very high correlation but the results were not significant (3 groups, r = 0.99).

### Niche model analysis

**Figure 7:**
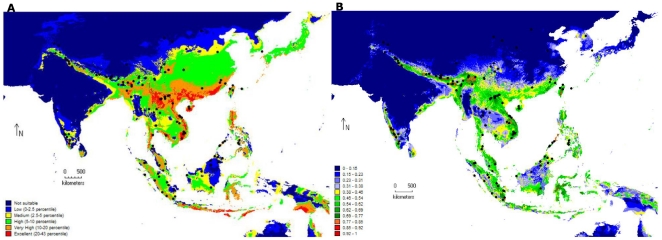
Niche model analysis with climatic data for leopard cats. Niche model analysis for the global population of leopard cats (*Prionailurus bengalensis*), predicting suitable versus unsuitable habitats using BIOCLIM and MaxEnt algorithms. Analyses included 140 unique records of leopard cat locations (black dots) obtained from museum records, literature and current study and 8 variables of temperature (annual range, maximum summer, mean of the driest and coldest quarter) and precipitation (wettest month, seasonality, warmest and coldest quarter). Climatic data was obtained from the WORLDCLIM data set [42] for 2.5 min intervals. **7A.** BIOCLIM model showing habitat suitability for leopard cats as a percentile of occurrences. Blue is unsuitable habitat, while red is excellent habitat. **7B.** MaxEnt model showing habitat suitability for leopard cats as a probability of occurrence where blue indicates very low probabilities and red indicates high probability.

Both approaches, BIOCLIM and MaxEnt had similar outputs for leopard cat and predicted the Central Indian region to be unsuitable habitat for the species, implying a break in geographical distribution (Figure 7). The mean value of the Area Under Curve (AUC) of the Receiver Operating Characteristic (ROC) for the test data of ten models using BIOCLIM was 71.2% (range: 62.8% to 75.2%). For the ten MaxEnt models the mean AUC for the training data was 88.5% (range: 87.7% to 90.2%) while for the test data, mean AUC was 83.8% (range: 79.8% to 87.1%).

**Figure 8:**
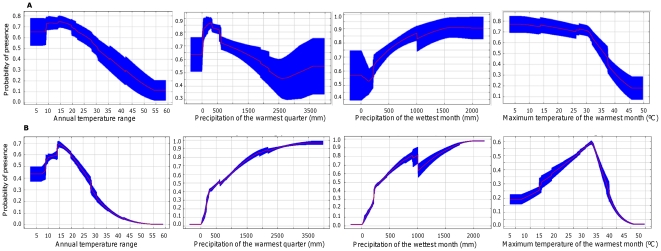
Response curves of MaxEnt models of habitat suitability for leopard cat (*Prionailurus bengalensis*) to predictor variables. **8A.** Models constructed by altering each variable at a time while keeping all others at their average values. Response curves presented as means of 10 replicates with standard deviation in blue. **8B.** Models constructed using only one variable at a time. Response curves presented as means of 10 replicates with standard deviation in blue.

Since BIOCLIM does not have a variable weighting function (since all variables are given equal weights), variable importance could only be determined from MaxEnt. The highest percentage contribution towards model fitting was from the variable, annual temperature range which had a contribution of 44.9% followed by the precipitation of the warmest quarter (14.7%), precipitation of the wettest month (13.9%), maximum temperature of the warmest month (8.3%), precipitation of the coldest quarter (7.8%), mean temperature of the coldest quarter (4.8%), precipitation seasonality (3.5%) and mean temperature of the driest quarter (2%).Jackknife tests showed Bio 7 to have the most useful information and Bio 18 to have information that other variables do not have. Bio 7 also contributed maximally towards the AUC estimate for the test data. Marginal response curves (models constructed using one variable at a time) were sharper for temperature variables as compared to precipitation (Figure 8). Both Bio 7 and Bio 5 showed sharp declines in marginal response curves at higher values (Bio 7 at temperature ranges above 15 and Bio 5 at approximately temperatures above 32°C However, a comparison of marginal response curves with response curves (models constructed by altering each variable at a time while keeping all others at their average values) reveals correlations and interactions between variables (Figure 8).

**Figure 9:**
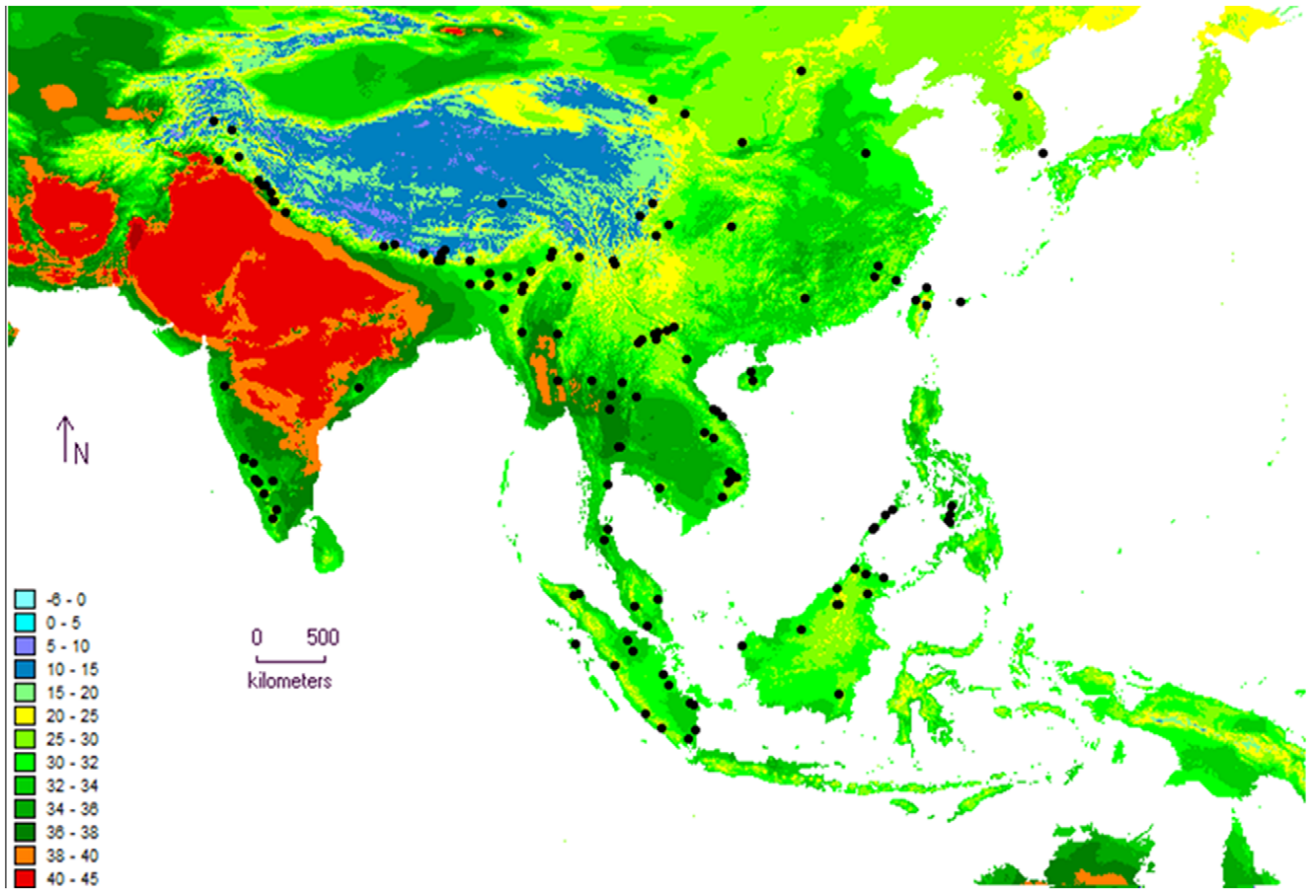
Map of Asia showing leopard cat locations superimposed over maximum temperatures in the warmest month. Leopard cat (*Prionailurus bengalensis*) locations (140 unique records) from across their global range were obtained from museum records, literature and current study. These locations are superimposed over maximum temperatures in the warmest month (°C), showing an upper threshold of 38°C, beyond which there are no records of leopard cat presence. Climatic data was obtained from the WORLDCLIM data set [42] for 2.5 min intervals. We used the software Diva-GIS (available at: http://www.diva-gis.org/climate; accessed on 4th April 2009) to construct the map.

Maps of various climatic variables with leopard cat locations show that the maximum temperature in the warmest month (Bio 5) explained leopard cat distribution most unambiguously and matched the predictions of the niche model best. An upper threshold of 35°C for the maximum temperatures in the warmest month was inferred from this map, beyond which leopard cat locations were very sparse (Figure 9). The mean value for Bio 5 associated with presence points was 29.47°C (95% Confidence Interval: 28.65°C to 30.29°C, n = 140). There are no records of leopard cats in regions where summer temperatures exceed 38°C. None of the other maps of climatic variables explained the distribution pattern as well.

## Discussion

Given their recent evolutionary history, broad distribution, vagile nature, and relatively similar ecologies, we expected that leopard cats and jungle cats would have similar patterns of genetic variation across the Indian subcontinent. However, our analyses revealed a stark difference in genetic variation and population structure between the two species. While the jungle cat, as predicted, shows high variation and significant but relatively low structure, the leopard cat is deeply structured into two populations. Most importantly, our results resolved the ambiguity surrounding leopard cat distribution in India by showing that the North and South Indian populations are not connected. Although the niche models show some very small patches of suitable habitat (albeit with very low suitability) for leopard cat in Central India, in and around Kanha Tiger Reserve, our sampling in Kanha did not yield any positive result for this species. Moreover, though some reports suggest the occurrence of leopard cat in Kanha, these are unauthenticated and there is no photographic evidence of the presence of this species there. Suitable habitats not occupied by the species can be explained by the inability of the species to reach or persist there due to barriers or inter-specific competition [49]. However, reports of the possible absence of leopard cats from Central and Western India do exist [15]–[17]. Despite the hypothesized absence, there was no mention of the two populations being disjunct and Pocock (1939) [16] even clumped the North-East Indian and South Indian populations as one, stating its distribution within India as ‘Peninsular India’. However, conventional field surveys cannot confirm absence since the inability to obtain positive records need not prove absence and the current distribution map provided by the Red List of Threatened Species [17] shows connectivity. Although we could not sample the Central Indian region intensively, we sampled areas that covered a wide range of habitats and climatic conditions (e.g. Kuno-Palpur to the west which was predominantly dry deciduous, Kanha which has a range of habitats from moist and dry deciduous forests to meadows, Nannaj which is predominantly agriculture and grassland). Leopard cats occupy a large range of habitats [13], [15], [41] and hence we believe our sampling was not biased against the species. We believe that the lack of specificity of habitat led many researchers and naturalists to extrapolate their range across peninsular India without any substantial evidence for doing so. Our results support earlier inferences of possible absence of leopard cats from Central India and further show the limits of distribution for this species within India with the Himalayan and the North-East Indian populations being more similar to each other than either of them is to the Western Ghats one. An additional support of our hypothesis comes from the total absence of any museum records of leopard cats from that region of India.

Although the *F*_ST_ values for jungle cat were relatively low (as compared to the leopard cat) they were significant and showed a low level of structuring which was not at all captured in the very shallow and unresolved phylogenetic tree and this could be a consequence of short and insufficient sequence lengths. On the other hand this was a comparative study and similar regions of DNA for leopard cat showed a contrasting pattern with strong phylogenetic separation between populations and significant population structuring. The pitfalls of using only mtDNA include effects of social organization such as female philopatry that could impact phylogeographic patterns, [50]–[52]. The occurrence of female philopatry in solitary, polygynous species has been documented and discussed widely [53]–[55], and has been demonstrated empirically in some cats [15], [56], [57] though not specifically in leopard cats and jungle cats. Leopard cat social organization follows the typical felid, polygynous system of one male holding a large territory encompassing several smaller female territories [15]. The jungle cat has not been studied from that perspective but since its close relatives (wild cat: *Felis silvestris* and black footed cat: *Felis nigripes*) [15], [56] follow the pattern it is very likely that jungle cat social organization adheres to the typical felid one, with no reason to believe otherwise. The expected similarity in social organization and dispersal along with similar body sizes for the two species discussed in our paper enables comparison despite using a one linked, maternally inherited marker.

Morphological plasticity or convergence in traits could possibly be the reason why the classical taxonomic grouping (which largely relied on morphological traits) did not perform as well in predicting genetic variation for the leopard cat. However, a combination of the three approaches explained the jungle cat pattern well.

In the case of the jungle cat, the biogeographic and taxonomic groupings showed similar results of significant but relatively low differentiation between populations (Tables 3 and 4). The desert population (Thar in the biogeographic approach or *F. c. prateri* in the taxonomic approach) and Upper Gangetic Plains/North East populations (*F. c. affinis*) showed high differentiation with all other groups. All three (biogeographic, taxonomic and latitudinal) approaches showed high differentiation between the Southern Indian (*F. c. kelaarti*, S. Deccan, latitudinal range: 10–19.9^o^ N) and the Upper Gangetic Plains/North East (*F. c. affinis*, Upper Gangetic Plains/North East, latitudinal range: 20–28.9^o^) populations, suggesting isolation by distance. From the biogeographic/ecoregion and the taxonomic approach it appears that the Central Indian population or *F. c. kutas* (which occurs throughout Central India) separates the Thar (*F. c. prateri*) from the rest and also the South Indian (*F. c. kelaarti*) populations from the Upper Gangetic Plains/North East (*F. c. affinis*). The Central Indian populations are genetically closer to the southern populations than to the northern, Upper Gangetic Plains/North East populations as shown by all the approaches.

Relatively low population structure, a shallow phylogenetic tree despite high genetic variation, a star-like network, θs>θπ, high negative and significant values of Fu's F and Tajima's D, and the unimodal mismatch fit (Figure 2, Figure 3 and Figure 6; Table 2 and Table 6) implicate population expansion for the jungle cat, which we suggest corresponds to a range expansion within India. This range expansion dates to the mid Pleistocene (166,709 to 271,342 years ago) based on the mismatch distribution. It is possible that the jungle cat colonized India from a dry, hot region to the west, and as suggested by the genetics does not face (either in the past or in present time) many barriers to its dispersal within the country (except in the higher altitudes of the Himalayas). However, *F*_ST_ values though relatively low are significant and some structuring is apparent, following an isolation by distance pattern. A study at finer scales and at the population level, using larger sequence lengths and nuclear data are required to identify factors contributing to this structure (social organization, competition, prey distribution, adaptation or physical barriers to dispersal) [58]. From natural history notes on its habitat and habits, it appears that this cat is limited by the combined availability of open habitats and water in the form of perennial water bodies [13], [15], [59]. Other ecological parameters such as competition from similar sized cats, along with genetic information from across its global range could perhaps further explain limits to its distribution.

In contrast, the leopard cat shows strong population structure, with the North Indian population separated from the Western Ghats one almost completely with an *F*_ST_ of 0.86 (Table 3). Such strong genetic difference suggests a break in their spatial distribution implying a barrier to their dispersal. From MaxEnt results and a visual interpretation of maps and response curves, the barrier appears to be influenced more by temperature than precipitation. MaxEnt picked out the annual temperature range (Bio 7) as the most influential variable describing leopard cat distribution while maps of each climatic variable showed maximum summer temperatures to be highly correlated to distribution limits. Although at the onset we selected variables that were not highly correlated to each other, a closer look at response curves (Figure 8) shows considerable interaction between variables and the effect of precipitation on distribution cannot be totally ignored. Maximum summer temperatures show a stable probability of presence that suddenly drops after around 32°C. Despite annual temperature ranges being very high in the higher latitudes (e.g. along the Himalayas), leopard cats do occur there and both models show the region to be suitable habitat. On the contrary, similar temperature ranges in lower latitudes around Central India show unsuitable habitat. Seemingly, leopard cats are less tolerant of wide annual temperature ranges especially in regions where summer temperatures are high. There are no records of leopard cats in regions that have summer temperatures above 38°C.Nevertheless, our results are correlational and hypothetical and there are likely to be other ecological variables such as competition and habitat alterations that could have a larger causal role in explaining leopard cat distribution.

These results have very strong conservation implications for the leopard cat. The IUCN currently recognizes the Indian population as one that is contiguous with the Asian mainland population and has categorized it as Least Concern, although it is listed in Appendix I of CITES due to the large illegal trade for its pelt [17]. The phylogenetic trees show the two populations within India to be reciprocally monophyletic. Since the leopard cat is also absent from Sri Lanka [15]–[17] our genetic data suggest that the Western Ghats population is effectively an island population, separated by a large geographic distance from any other leopard cat population. Further analyses with autosomal markers (microsatellites) are required to authenticate these inferences. The haplotype network reveals only two haplotypes for this island population. This “pruned” genetic variation could be the result of a bottleneck due to climatic change and extinctions (invoking vicariance) or a founder effect (dispersal and colonization). Irrespective of what caused low variation in this population, the fact that it is geographically isolated and harbors low variation strongly advocates special conservation attention for southern Indian leopard cats.

Although the niche-modelling now allows us to hypothesize a currently disjunct distribution for the leopard cat, it is still not known when, where and why the break came about. Through vegetation studies it has been inferred that during the Last Glacial Maximum (LGM: approximately 23,000 to 18,000 years BP) large parts of India and Pakistan became more arid and hot [60]–[64], suggesting a role for this climatic event in the genetic pattern we observe for leopard cats.

Based on our results and inferences from niche modeling, we attempt to explain our genetic data on leopard cats through vicariance. The leopard cat may have come into India from the east and in the past would have occurred all through the cooler parts of India. The phylogenetic tree and haplotype network for the global population of leopard cat, though unresolved, show a link between the Thailand and Indian populations. However, due to the effects of the Quaternary glaciations (drying and heating up of the subcontinent) they might have retreated to refugia, explaining the current distribution. Though debatable, this explanation has been offered for many mammalian species that have distributions restricted to the Himalayas and Western Ghats, such as the tahr (Himalayan: *Hemitragus jemlahicus*, Nilgiri: *Nilgiritragus hylocrius*) and marten (Himalayan: *Martes foina*, Nilgiri: *Martes gwatkinsii*) [10]. Such a historical scenario would also suggest that leopard cats moved into South India after the bridge between Sri Lanka and India ceased to exist, approximately 15,000 years ago [16] and hence could not cross over to Sri Lanka. The divergence between the North Indian and South Indian leopard cat clades would then have to be relatively recent (less than 15000 years). However, the observed genetic differences between the North Indian and South Indian leopard cat populations suggest a potentially longer-term separation. Greater sequence length and nuclear markers such as microsatellites and samples from populations in Orissa (India) [65], Myanmar, South China and Thailand (that were not covered in the current or past study [23]) and time to most recent common ancestor (TMRCA) calculations would allow us to quantify the divergence time between these populations. Alternatively missing haplotypes shown in the network could have been lost due to evolutionary processes like lineage sorting or population isolation followed by drift. It is also possible that the leopard cat did cross over to Sri Lanka but was not able to establish itself or persist there due to an earlier presence of other similar sized cats such as the jungle cat and the rusty spotted cat (*Prionailurus rubiginosa*).

Several island populations of leopard cats are known to occur and have been assigned sub-specific status [17], [23]. However, the presence of an island population within a mainland is interesting. Such distributions have been reported for other felids e.g. fishing cat (*Prionailurus viverrinus*) [13]. Vicariance due to topographical features are often sought to explain breaks in species distributions. However, for many larger bodied species, barriers may not be obvious unless global distributions are taken into account. In our study a climatic barrier, suitably explains the adaptive potential, distribution and genetic variation of an otherwise common species. Our study reiterates the view that a comparison across related species with seemingly similar requirements not only brings out the biogeographic history of the region but also important details of adaptive thresholds and current barriers to dispersal. Furthermore, the distribution of the leopard cat in India was not clear until combined results of historical records, genetic data and niche modeling showed a clear break in their distribution.

In this paper, we show that two seemingly similar species have strikingly different phylogeographic patterns. Further, we suggest that these differences could be due to interactions between habitat preference and climatic transitions in the past. The Indian subcontinent supports a variety of habitats with a complex geological and paleoclimatic history. Our results underscore the point that given this finding a single paradigm to explain patterns of genetic diversity in this region, in the manner that the glaciations have for Europe and North America [4], [5], [12], might prove difficult. Our study does indicate that ecological thresholds (climatic, physiological, habitat) and the strength of these thresholds in limiting and restricting distributions are perhaps good predictors of genetic variation and structuring. Although ecological thresholds are complex and difficult to estimate, proxies combining climatic and habitat variables (wet/dry, cold/hot, open/closed) that describe a species range, could perhaps be good indicators of thresholds, for larger bodied mammals. Additionally, given India's location at the confluence of major biogeographic realms, understanding phylogeographic patterns in this region might help predict patterns of genetic variation and the impact of species ecology on such variation for widely distributed species elsewhere. Such studies will not only enhance our understanding of specific species, but also contribute to a deeper understanding of the relative importance of species ecology and evolutionary history in determining present distributions, and possibly allow prediction of future responses of species to changing environments.

## Supporting Information

Table S1 Sample identities with accession numbers and localities.
(0.15 MB DOC)
