# Supplementary material for: Ecology Driving Genetic Variation: A Comparative Phylogeography of Jungle Cat (Felis chaus) and Leopard Cat (Prionailurus bengalensis) in India
Source: PLoS One. 2010 Oct 29;5(10):e13724. doi: 10.1371/journal.pone.0013724 (PMC2966403; doi:10.1371/journal.pone.0013724)
Supplement: Table S1 — Sample identities with accession numbers and localities. (0.15 MB DOC) [file pone.0013724.s001.doc]

**Table S1.** Sample identities with accession numbers and localities.

| ID | Accession Number NADH5 | Accession Number Cytochrome b | Species | State | Biogeographic zone |
| --- | --- | --- | --- | --- | --- |
| JCTVM3 | GU561646 | GU561701 | Jungle cat | Trivandrum zoo, Kerala | Deccan South |
| JCMyso | GU561647 | GU561702 | Jungle cat | Mysore zoo, Karnataka | Deccan South |
| JCAP04 | GU561648 | GU561703 | Jungle cat | Andhra Pradesh (AP) | Deccan South |
| JCAP02 | GU561649 | GU561704 | Jungle cat | AP | Deccan South |
| JCA001 | GU561650 | GU561705 | Jungle cat | Kerala | Western Ghats |
| JCA008 | GU561651 | GU561706 | Jungle cat | Kerala | Western Ghats |
| JCEKM3 | GU561652 | GU561707 | Jungle cat | Kerala | Western Ghats |
| JCKEM1 | GU561653 | GU561708 | Jungle cat | Kerala | Western Ghats |
| JC007C | GU561654 | GU561709 | Jungle cat | Kerala | Western Ghats |
| JcTVM2 | GU561655 | GU561710 | Jungle cat | Trivandrum zoo, Kerala | Deccan South |
| JCMAND | GU561656 | GU561711 | Jungle cat | Karnataka | Deccan South |
| JCHZ01 | GU561657 | GU561712 | Jungle cat | Hyderabad zoo, AP | Deccan South |
| JCHZ03 | GU561658 | GU561713 | Jungle cat | Hyderabad zoo, AP | Deccan South |
| JCN001 | GU561659 | GU561714 | Jungle cat | Maharashtra | Deccan Central |
| JCN004 | GU561660 | GU561715 | Jungle cat | Maharashtra | Deccan Central |
| JCN005 | GU561661 | GU561716 | Jungle cat | Maharashtra | Deccan Central |
| JCN002 | GU561662 | GU561717 | Jungle cat | Maharashtra | Deccan Central |
| JCNS10 | GU561663 | GU561718 | Jungle cat | Maharashtra | Deccan Central |
| JCNSC8 | GU561664 | GU561719 | Jungle cat | Maharashtra | Deccan Central |
| JCNSC9 | GU561665 | GU561720 | Jungle cat | Maharashtra | Deccan Central |
| JCN003 | GU561666 | GU561721 | Jungle cat | Maharashtra | Deccan Central |
| JCNaS1 | GU561667 | GU561722 | Jungle cat | Maharashtra | Western Ghats |
| JCkatr | GU561668 | GU561723 | Jungle cat | Maharashtra | Western Ghats |
| JCNS11 | GU561669 | GU561724 | Jungle cat | Maharashtra | Deccan Central |
| JCWB02 | GU561670 | GU561725 | Jungle cat | West Bengal (WB) | Lower Gangetic Plain |
| JCWB01 | GU561671 | GU561726 | Jungle cat | WB | Lower Gangetic Plain |
| JCCORB | GU561672 | GU561727 | Jungle cat | Uttarakhand | Upper Gangetic Plain |
| JCHG01 | GU561673 | GU561728 | Jungle cat | Gujarat | Semi Arid |
| JCSTR023 | GU561674 | GU561729 | Jungle cat | Rajasthan | Semi Arid |
| JCSTR014 | GU561675 | GU561730 | Jungle cat | Rajasthan | Semi Arid |
| JSL057 | GU561676 | GU561731 | Jungle cat | Rajasthan | Thar Desert |
| JCBKN023 | GU561677 | GU561732 | Jungle cat | Rajasthan | Thar Desert |
| JCBKN001 | GU561678 | GU561733 | Jungle cat | Rajasthan | Thar Desert |
| JCBKN004 | GU561679 | GU561734 | Jungle cat | Rajasthan | Thar Desert |
| JCBKN018 | GU561680 | GU561735 | Jungle cat | Rajasthan | Thar Desert |
| JCBKN034 | GU561681 | GU561736 | Jungle cat | Rajasthan | Thar Desert |
| JCRAJ1 | GU561682 | GU561737 | Jungle cat | Rajasthan | Thar Desert |
| JCKU13 | GU561683 | GU561738 | Jungle cat | Madhya Pradesh (MP) | Semi Arid |
| JCKU30 | GU561684 | GU561739 | Jungle cat | MP | Semi Arid |
| JCKU26 | GU561685 | GU561740 | Jungle cat | MP | Semi Arid |
| JCKU09 | GU561686 | GU561741 | Jungle cat | MP | Semi Arid |
| JCKTR1 | GU561687 | GU561742 | Jungle cat | MP | Deccan Central |
| JCRaj2 | GU561688 | GU561743 | Jungle cat | Rajasthan | Thar Desert |
| JCRaj3 | GU561689 | GU561744 | Jungle cat | Rajasthan | Thar Desert |
| JCSTR051 | GU561690 | GU561745 | Jungle cat | Rajasthan | Semi Arid |
| JCORS001 | GU561691 | GU561746 | Jungle cat | Rajasthan | Deccan Central |
| JCGZ03 | GU561692 | GU561747 | Jungle cat | Guwahati zoo, Assam | North East |
| JCGZ02 | GU561693 | GU561748 | Jungle cat | Guwahati zoo, Assam | North East |
| JCGZ04 | GU561694 | GU561749 | Jungle cat | Guwahati zoo, Assam | North East |
| JCGZ14 | GU561695 | GU561750 | Jungle cat | Guwahati zoo, Assam | North East |
| JCGZ15 | GU561696 | GU561751 | Jungle cat | Guwahati zoo, Assam | North East |
| JCGZ16 | GU561697 | GU561752 | Jungle cat | Guwahati zoo, Assam | North East |
| JCAIZ4 | GU561698 | GU561753 | Jungle cat | Aizawl zoo, Mizoram | North East |
| JCGZ01 | GU561699 | GU561754 | Jungle cat | Guwahati zoo, Assam | North East |
| JCAIZ003 | GU561700 | GU561755 | Jungle cat | Aizawl zoo, Mizoram | North East |
| LCZKS1 | GU561756 | GU561800 | Leopard cat | Jammu& Kashmir (J&K) | Himalayas |
| LCS4 | GU561757 | GU561801 | Leopard cat | Sikkim | Himalayas |
| LCS5 | GU561758 | GU561802 | Leopard cat | Sikkim | Himalayas |
| LCS6 | GU561759 | GU561803 | Leopard cat | Sikkim | Himalayas |
| LCS7 | GU561760 | GU561804 | Leopard cat | Sikkim | Himalayas |
| LCS12 | GU561761 | GU561805 | Leopard cat | Sikkim | Himalayas |
| LCS9 | GU561762 | GU561806 | Leopard cat | Sikkim | Himalayas |
| LCNam3 | GU561763 | GU561799 | Leopard cat | Arunachal Pradesh (ArP) | Himalayas |
| LCNam6 | GU561764 | GU561808 | Leopard cat | ArP | Himalayas |
| LCHP44 | GU561765 | GU561809 | Leopard cat | Himachal Pradesh (HP) | Himalayas |
| LCHP45 | GU561766 | GU561810 | Leopard cat | HP | Himalayas |
| LCGZ10 | GU561767 | GU561811 | Leopard cat | Guwahati zoo, Assam | North East |
| LCDG5 | GU561768 | GU561812 | Leopard cat | J&K | Himalayas |
| LCHP3 | GU561769 | GU561813 | Leopard cat | HP | Himalayas |
| LCGZ6 | GU561770 | GU561814 | Leopard cat | Guwahati zoo, Assam | North East |
| LCGZ8 | GU561771 | GU561798 | Leopard cat | Guwahati zoo, Assam | North East |
| LCGZ9 | GU561772 | GU561816 | Leopard cat | Guwahati zoo, Assam | North East |
| LCDG13 | GU561773 | GU561817 | Leopard cat | J&K | Himalayas |
| LCDG10 | GU561774 | GU561818 | Leopard cat | J&K | Himalayas |
| LCAIZ2 | GU561775 | GU561820 | Leopard cat | Aizawl zoo, Mizoram | North East |
| LCDARZ1 | GU561776 | GU561821 | Leopard cat | Darjeeling zoo, WB | North East |
| LCDARZ2 | GU561777 | GU561822 | Leopard cat | Darjeeling zoo, WB | North East |
| LCBRH6 | GU561778 | GU561823 | Leopard cat | Karnataka | Western Ghats |
| LCBhad6 | GU561779 | GU561824 | Leopard cat | Karnataka | Western Ghats |
| LCPTR1 | GU561780 | GU561825 | Leopard cat | Kerala | Western Ghats |
| LCPTR2 | GU561781 | GU561797 | Leopard cat | Kerala | Western Ghats |
| LCBRH3 | GU561782 | GU561796 | Leopard cat | Karnataka | Western Ghats |
| LCSV5 | GU561783 | GU561826 | Leopard cat | Kerala | Western Ghats |
| LCBHAD1 | GU561785 | GU561827 | Leopard cat | Karnataka | Western Ghats |
| LCBHAD2 | GU561786 | GU561828 | Leopard cat | Karnataka | Western Ghats |
| LCBHD3 | GU561787 | GU561829 | Leopard cat | Karnataka | Western Ghats |
| LCBhad5 | GU561788 | GU561830 | Leopard cat | Karnataka | Western Ghats |
| LCCHK8 | GU561789 | GU561832 | Leopard cat | Karnataka | Western Ghats |
| LCCHK3 | GU561790 | GU561831 | Leopard cat | Karnataka | Western Ghats |
| LCGZ5 | GU561791 | GU561835 | Leopard cat | Guwahati zoo, Assam | North East |
| LCDG11 | GU561792 | GU561836 | Leopard cat | J&K | Himalayas |
| LCHP4 | GU561793 | GU561837 | Leopard cat | HP | Himalayas |
| LCHP48 | GU561794 | GU561834 | Leopard cat | HP | Himalayas |
| LCHP18 | GU561795 | GU561833 | Leopard cat | HP | Himalayas |
| LC AIZ1 | GU561784 | GU561819 | Leopard cat | Aizawl zoo, Mizoram | North East |
